# 3-Benzyl-5,7-dimeth­oxy­chroman-4-ol

**DOI:** 10.1107/S1600536811002066

**Published:** 2011-02-23

**Authors:** Mahidansha M. Shaikh, Glenn E.M. Maguire, Hendrik G. Kruger, Karen du Toit

**Affiliations:** a School of Pharmacy and Pharmacology, University of KwaZulu-Natal, Durban 4000, South Africa; b School of Chemistry, University of KwaZulu-Natal, Durban 4000, South Africa

## Abstract

In the crystal structure of the title compound, C_18_H_20_O_4_, O—H⋯O hydrogen bonds connect the mol­ecules in parallel layers along the *b* axis.

## Related literature

For analogous structures, see Koch *et al.* (1994 ▶); Porter *et al.* (1985 ▶). For the biological activity of naturally ocurring homoisoflavanones that possess a 3-benzyl-substituted chroman ring system, see: Zhang *et al.* (2008 ▶). For our work on the synthesis and characterization of natural products from this family of compounds in the search for new medical agents, see: Shaikh *et al.* (2011 ▶).
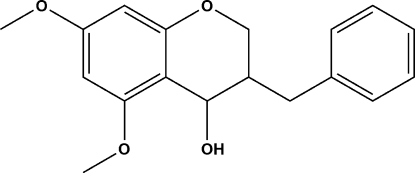


## Experimental

### 

#### Crystal data

C_18_H_20_O_4_
*M* *_r_* = 300.34
Monoclinic, 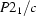

*a* = 9.870 (5) Å
*b* = 11.211 (6) Å
*c* = 14.603 (7) Å
β = 107.072 (7)°
*V* = 1544.6 (13) Å^3^
*Z* = 4
Mo *K*α radiation
μ = 0.09 mm^−1^
*T* = 100 K
0.37 × 0.24 × 0.20 mm

#### Data collection

Bruker Kappa DUO APEXII diffractometer
12055 measured reflections
3882 independent reflections
3369 reflections with *I* > 2σ(*I*)
*R*_int_ = 0.021

#### Refinement

*R*[*F* ^2^ > 2σ(*F* ^2^)] = 0.037
*wR*(*F* ^2^) = 0.100
*S* = 1.04
3882 reflections
203 parameters
1 restraint
H atoms treated by a mixture of independent and constrained refinement
Δρ_max_ = 0.38 e Å^−3^
Δρ_min_ = −0.21 e Å^−3^

### 

Data collection: *APEX2* (Bruker, 2006 ▶); cell refinement: *SAINT* (Bruker, 2006 ▶); data reduction: *SAINT*; program(s) used to solve structure: *SHELXS97* (Sheldrick, 2008 ▶); program(s) used to refine structure: *SHELXL97* (Sheldrick, 2008 ▶); molecular graphics: *OLEX2* (Dolomanov *et al.*, 2009 ▶); software used to prepare material for publication: *SHELXL97*.

## Supplementary Material

Crystal structure: contains datablocks I, global. DOI: 10.1107/S1600536811002066/pb2047sup1.cif

Structure factors: contains datablocks I. DOI: 10.1107/S1600536811002066/pb2047Isup2.hkl

Additional supplementary materials:  crystallographic information; 3D view; checkCIF report

## Figures and Tables

**Table 1:** Hydrogen-bond geometry (Å, °)

*D*—H⋯*A*	*D*—H	H⋯*A*	*D*⋯*A*	*D*—H⋯*A*
O2—H2*O*⋯O1^i^	0.95 (1)	1.93 (1)	2.8366 (15)	158 (2)

Symmetry code: (i) 
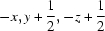
.

**Fig. 1.**
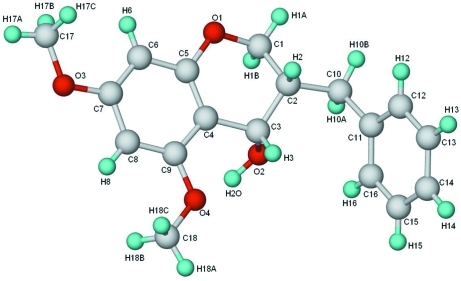
Molecular structue of the title compound showing the numbering scheme.

**Fig. 2.**
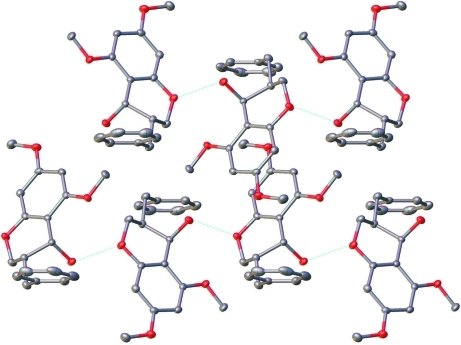
Projection viewed along [100]. All hydrogen have been omitted for clarity. The hydrogen bonds are shown as dotted lines.

## supplementary crystallographic information

### Comment

Naturally ocurring homoisoflavanones that posses a 3-benzyl-substituted chroman ring system as a common framework have been isolated from a wide range of natural sources and exhibit a variety of biological activities (Zhang *et al.*, 2008). We recently have been involved in the synthesis and characterization of natural products from this family of compounds in the search for new medical agents (Shaikh *et al.*, 2011). The title compound is an intermediate step in the synthesis of 5,7 dimethoxy-3-benzyl-4-chroman-none.

There a few analogous structures of chroman alcohols bearing a benzyl ring found in the literature. The two closest have the 5,7 dimethoxy moieties, where one is a biphenyl derivative with an alkylated ketone at the 4 position (Koch *et al.*,1994) the other has a phenyl group at the 2 position but no alcohol functionality (Porter *et al.*, 1985). Here we report the first example where a chroman-ol benzyl derivative (Fig. 1) that demonstrates hydrogen bonding in the solid state. This intermolecular hydrogen bond O2—H—O1 (2.8366 Å) holds the structure in two parallel plains (Fig. 2). The intermolecular distances between the ring centroids are all greater than 6 Å suggesting that there is no π -stacking.

### Experimental

To a solution of 5,7-dimethoxy-3-(benzyl)-4-chromanone (1.0 g, 3.3 mmol) in anhydrous MeOH (15 ml), NaBH_4_ (0.38 g, 10.0 mmol) was added portionwise at a temperature of 0 °C under a nitrogen atmosphere. The mixture was then allowed to reach room temperature and stirred for 1 h. The reaction mixture was quenched with water and extracted with ethyl acetate (3 *x* 30 ml). The organic layer was washed with brine, dried over magnesium sulfate, and concentrated under reduced pressure to produce a viscous oil mixture. The residue obtained after evaporation of the solvent was chromatographed over a silica gel column using mixture of ethyl acetate/hexane (30:70) as eluent product to yield of 88% (0.88 g). Off-white solid; m.p. 118–121 °C. The title compound was recrystalized from a solution of ethyl acetate/hexane (30:70) at room temperature.

^1^H NMR (400 MHz, CDCl_3_, δ, p.p.m.): 7.33–7.26 (m, 5H), 6.02 (d, *J*=2.20 Hz, 1H), 6.00 (d, *J*=2.20 Hz, 1H), 4.70 (d, *J*=2.40 Hz, 1H), 4.02 (dd, *J*=3.68, 6.20 Hz, 2H), 3.77 (s, 3H), 3.73 (s, 3H), 2.95 (dd, *J*=8.08, 8.12 Hz, 1H), 2.66 (dd, *J*=7.44, 7.44 Hz, 1H).

^13^C NMR (100 MHz, CDCl_3_, δ, p.p.m.): 161.1, 159.2, 155.9, 139.6, 129.1, 128.4, 126.1, 106.7, 93.0, 91.4, 65.2, 59.6, 55.4, 55.3, 40.0, 32.9.

IR: 3501, 2946, 1592, 1453, 1304, 1200, 1052.

HRMS (EI): Calcd for C_18_H_20_O_4_Na 323.1254, found 323.1271.

### Refinement

Single-crystal X-ray diffraction data were collected on a Bruker *KAPPA APEX* II DUO diffractometer using graphite-monochromated Mo—Ka radiation (c = 0.71073 Å). Data collection was carried out at 100 (2) K. Temperature was controlled by an Oxford Cryostream cooling system (Oxford Cryostat). Cell refinement and data reduction were performed using the program *SAINT* (Bruker, 2006). The data were scaled and empirical absorption corrections were performed using *SADABS* (Sheldrick, 1997). The structure was solved by direct methods using *SHELXS97* (Sheldrick, 2008) and refined by full-matrix least-squares methods based on F^2^ using *SHELXL97* (Sheldrick, 2008) and using the graphics interface program *X-SEED* (Barbour, 2001). All non-hydrogen atoms were refined anisotropically. All hydrogen atoms, except the hydroxyl hydrogen, were positioned geometrically with C—H distances ranging from 0.95 Å to 1.00 Å and refined as riding on their parent atoms, with *U*_iso_ (H) = 1.2 - 1.5 *U*_eq_ (C).

### Crystal data

**Table:**

C_18_H_20_O_4_	1544.6(13)
*M**_r_* = 300.34	*D*_x_ = 1.292 Mg m^−^^3^
Monoclinic, *P*2_1_/*c*	Mo *K*α radiation, λ = 0.71073 Å
*a* = 9.870 (5) Å	Cell parameters from 12055 reflections
*b* = 11.211 (6) Å	θ = 2.2–28.5°
*c* = 14.603 (7) Å	µ = 0.09 mm^−^^1^
β = 107.072 (7)°	*T* = 100 K
*V* = 1544.6 (13) Å^3^	Needle, colourless
*Z* = 4	0.37 × 0.24 × 0.20 mm
*F*(000) = 640	

### Data collection

**Table:**

Bruker Kappa DUO APEXII diffractometer	3369 reflections with *I* > 2σ(*I*)
Radiation source: fine-focus sealed tube	*R*_int_ = 0.021
graphite	θ_max_ = 28.5°, θ_min_ = 2.2°
0.5° φ scans and ω scans	*h* = −12→13
12055 measured reflections	*k* = −15→14
3882 independent reflections	*l* = −19→9

### Refinement

**Table:**

Refinement on *F*^2^	Primary atom site location: structure-invariant direct methods
Least-squares matrix: full	Secondary atom site location: difference Fourier map
*R*[*F*^2^ > 2σ(*F*^2^)] = 0.037	Hydrogen site location: inferred from neighbouring sites
*wR*(*F*^2^) = 0.100	H atoms treated by a mixture of independent and constrained refinement
*S* = 1.04	*w* = 1/[σ^2^(*F*_o_^2^) + (0.0499*P*)^2^ + 0.5109*P*] where *P* = (*F*_o_^2^ + 2*F*_c_^2^)/3
3882 reflections	(Δ/σ)_max_ < 0.001
203 parameters	Δρ_max_ = 0.38 e Å^−^^3^
1 restraint	Δρ_min_ = −0.21 e Å^−^^3^

### Special details

**Table:**

Geometry. All e.s.d.'s (except the e.s.d. in the dihedral angle between two l.s. planes) are estimated using the full covariance matrix. The cell e.s.d.'s are taken into account individually in the estimation of e.s.d.'s in distances, angles and torsion angles; correlations between e.s.d.'s in cell parameters are only used when they are defined by crystal symmetry. An approximate (isotropic) treatment of cell e.s.d.'s is used for estimating e.s.d.'s involving l.s. planes.
Refinement. Refinement of *F*^2^ against ALL reflections. The weighted *R*-factor *wR* and goodness of fit *S* are based on *F*^2^, conventional *R*-factors *R* are based on *F*, with *F* set to zero for negative *F*^2^. The threshold expression of *F*^2^ > σ(*F*^2^) is used only for calculating *R*-factors(gt) *etc*. and is not relevant to the choice of reflections for refinement. *R*-factors based on *F*^2^ are statistically about twice as large as those based on *F*, and *R*- factors based on ALL data will be even larger.

### Fractional atomic coordinates and isotropic or equivalent isotropic displacement parameters (Å^2^)

**Table:**

	*x*	*y*	*z*	*U*_iso_*/*U*_eq_	
O1	−0.00588 (8)	−0.11619 (7)	0.23516 (5)	0.01815 (17)	
O2	0.16950 (8)	0.17225 (7)	0.31067 (5)	0.01963 (17)	
H2O	0.1135 (16)	0.2376 (12)	0.2790 (11)	0.046 (5)*	
O3	−0.32946 (8)	0.01390 (7)	−0.06048 (5)	0.02129 (18)	
O4	0.08477 (8)	0.24224 (7)	0.08733 (5)	0.01719 (16)	
C1	0.10969 (11)	−0.08620 (10)	0.31876 (7)	0.0177 (2)	
H1A	0.1427	−0.1592	0.3569	0.021*	
H1B	0.0757	−0.0295	0.3591	0.021*	
C2	0.23245 (10)	−0.03074 (9)	0.29118 (7)	0.0150 (2)	
H2	0.2580	−0.0855	0.2448	0.018*	
C3	0.18241 (10)	0.08729 (9)	0.23985 (7)	0.01346 (19)	
H3	0.2548	0.1163	0.2094	0.016*	
C4	0.04340 (10)	0.06766 (9)	0.16310 (7)	0.01331 (19)	
C5	−0.04198 (10)	−0.03082 (9)	0.16389 (7)	0.0144 (2)	
C6	−0.16888 (11)	−0.05346 (9)	0.09140 (7)	0.0165 (2)	
H6	−0.2252	−0.1213	0.0942	0.020*	
C7	−0.20927 (10)	0.02633 (10)	0.01570 (7)	0.0162 (2)	
C8	−0.12787 (11)	0.12749 (9)	0.01173 (7)	0.0163 (2)	
H8	−0.1576	0.1821	−0.0400	0.020*	
C9	−0.00293 (10)	0.14667 (9)	0.08470 (7)	0.01417 (19)	
C10	0.36316 (11)	−0.01853 (10)	0.37985 (7)	0.0185 (2)	
H10A	0.3408	0.0374	0.4259	0.022*	
H10B	0.3851	−0.0972	0.4116	0.022*	
C11	0.49155 (11)	0.02629 (10)	0.35418 (7)	0.0177 (2)	
C12	0.58336 (12)	−0.05326 (11)	0.32883 (8)	0.0232 (2)	
H12	0.5668	−0.1366	0.3300	0.028*	
C13	0.69930 (12)	−0.01154 (14)	0.30172 (9)	0.0313 (3)	
H13	0.7614	−0.0666	0.2851	0.038*	
C14	0.72403 (13)	0.11021 (14)	0.29902 (9)	0.0343 (3)	
H14	0.8024	0.1387	0.2800	0.041*	
C15	0.63369 (13)	0.18964 (13)	0.32419 (9)	0.0307 (3)	
H15	0.6504	0.2730	0.3225	0.037*	
C16	0.51859 (12)	0.14850 (11)	0.35193 (8)	0.0221 (2)	
H16	0.4578	0.2040	0.3695	0.027*	
C17	−0.40947 (12)	−0.09390 (11)	−0.06520 (8)	0.0237 (2)	
H17A	−0.4920	−0.0922	−0.1223	0.036*	
H17B	−0.4413	−0.1006	−0.0079	0.036*	
H17C	−0.3497	−0.1626	−0.0687	0.036*	
C18	0.05036 (12)	0.31979 (10)	0.00542 (8)	0.0204 (2)	
H18A	0.1204	0.3841	0.0158	0.031*	
H18B	−0.0441	0.3540	−0.0037	0.031*	
H18C	0.0512	0.2742	−0.0516	0.031*	

### Atomic displacement parameters (Å^2^)

**Table:**

	*U*^11^	*U*^22^	*U*^33^	*U*^12^	*U*^13^	*U*^23^
O1	0.0173 (3)	0.0177 (4)	0.0169 (4)	−0.0036 (3)	0.0012 (3)	0.0048 (3)
O2	0.0239 (4)	0.0178 (4)	0.0151 (3)	0.0049 (3)	0.0025 (3)	−0.0040 (3)
O3	0.0182 (4)	0.0241 (4)	0.0173 (4)	−0.0025 (3)	−0.0014 (3)	−0.0001 (3)
O4	0.0204 (4)	0.0153 (4)	0.0148 (3)	−0.0021 (3)	0.0037 (3)	0.0037 (3)
C1	0.0165 (4)	0.0200 (5)	0.0151 (4)	−0.0024 (4)	0.0022 (4)	0.0045 (4)
C2	0.0148 (4)	0.0150 (5)	0.0150 (4)	0.0008 (4)	0.0043 (4)	0.0013 (4)
C3	0.0149 (4)	0.0139 (5)	0.0116 (4)	−0.0004 (3)	0.0039 (3)	−0.0005 (3)
C4	0.0143 (4)	0.0144 (5)	0.0116 (4)	0.0012 (4)	0.0043 (3)	−0.0006 (3)
C5	0.0158 (4)	0.0149 (5)	0.0132 (4)	0.0018 (4)	0.0056 (4)	0.0012 (4)
C6	0.0154 (4)	0.0171 (5)	0.0172 (5)	−0.0016 (4)	0.0053 (4)	−0.0005 (4)
C7	0.0146 (4)	0.0198 (5)	0.0132 (4)	0.0010 (4)	0.0028 (4)	−0.0028 (4)
C8	0.0189 (5)	0.0169 (5)	0.0125 (4)	0.0023 (4)	0.0038 (4)	0.0016 (4)
C9	0.0169 (4)	0.0132 (4)	0.0136 (4)	0.0008 (4)	0.0062 (4)	−0.0006 (4)
C10	0.0165 (5)	0.0206 (5)	0.0169 (5)	0.0002 (4)	0.0025 (4)	0.0045 (4)
C11	0.0146 (4)	0.0221 (5)	0.0133 (4)	−0.0010 (4)	−0.0009 (4)	0.0021 (4)
C12	0.0199 (5)	0.0253 (6)	0.0217 (5)	0.0031 (4)	0.0020 (4)	0.0013 (4)
C13	0.0180 (5)	0.0516 (8)	0.0235 (6)	0.0070 (5)	0.0047 (4)	0.0035 (5)
C14	0.0173 (5)	0.0596 (9)	0.0227 (6)	−0.0095 (6)	0.0006 (4)	0.0103 (6)
C15	0.0269 (6)	0.0346 (7)	0.0238 (6)	−0.0143 (5)	−0.0033 (5)	0.0066 (5)
C16	0.0212 (5)	0.0226 (6)	0.0187 (5)	−0.0031 (4)	−0.0003 (4)	0.0004 (4)
C17	0.0187 (5)	0.0257 (6)	0.0234 (5)	−0.0043 (4)	0.0011 (4)	−0.0040 (4)
C18	0.0269 (5)	0.0175 (5)	0.0169 (5)	−0.0009 (4)	0.0066 (4)	0.0051 (4)

### Geometric parameters (Å, °)

**Table:**

O1—C5	1.3815 (13)	C8—C9	1.3895 (14)
O1—C1	1.4445 (13)	C8—H8	0.9500
O2—C3	1.4389 (13)	C10—C11	1.5091 (15)
O2—H2O	0.952 (9)	C10—H10A	0.9900
O3—C7	1.3746 (13)	C10—H10B	0.9900
O3—C17	1.4342 (15)	C11—C12	1.3966 (16)
O4—C9	1.3708 (13)	C11—C16	1.3981 (17)
O4—C18	1.4365 (13)	C12—C13	1.3967 (18)
C1—C2	1.5176 (15)	C12—H12	0.9500
C1—H1A	0.9900	C13—C14	1.389 (2)
C1—H1B	0.9900	C13—H13	0.9500
C2—C3	1.5296 (15)	C14—C15	1.384 (2)
C2—C10	1.5423 (15)	C14—H14	0.9500
C2—H2	1.0000	C15—C16	1.3919 (17)
C3—C4	1.5113 (14)	C15—H15	0.9500
C3—H3	1.0000	C16—H16	0.9500
C4—C5	1.3911 (15)	C17—H17A	0.9800
C4—C9	1.4131 (14)	C17—H17B	0.9800
C5—C6	1.4053 (15)	C17—H17C	0.9800
C6—C7	1.3864 (15)	C18—H18A	0.9800
C6—H6	0.9500	C18—H18B	0.9800
C7—C8	1.4009 (16)	C18—H18C	0.9800
			
C5—O1—C1	116.13 (8)	O4—C9—C4	114.56 (9)
C3—O2—H2O	108.8 (10)	C8—C9—C4	121.84 (9)
C7—O3—C17	117.16 (9)	C11—C10—C2	112.17 (9)
C9—O4—C18	117.17 (8)	C11—C10—H10A	109.2
O1—C1—C2	111.41 (9)	C2—C10—H10A	109.2
O1—C1—H1A	109.3	C11—C10—H10B	109.2
C2—C1—H1A	109.3	C2—C10—H10B	109.2
O1—C1—H1B	109.3	H10A—C10—H10B	107.9
C2—C1—H1B	109.3	C12—C11—C16	118.44 (11)
H1A—C1—H1B	108.0	C12—C11—C10	120.71 (10)
C1—C2—C3	108.44 (8)	C16—C11—C10	120.81 (10)
C1—C2—C10	110.47 (9)	C11—C12—C13	120.71 (12)
C3—C2—C10	113.88 (9)	C11—C12—H12	119.6
C1—C2—H2	108.0	C13—C12—H12	119.6
C3—C2—H2	108.0	C14—C13—C12	120.16 (12)
C10—C2—H2	108.0	C14—C13—H13	119.9
O2—C3—C4	112.14 (8)	C12—C13—H13	119.9
O2—C3—C2	107.73 (8)	C15—C14—C13	119.50 (12)
C4—C3—C2	109.25 (8)	C15—C14—H14	120.3
O2—C3—H3	109.2	C13—C14—H14	120.3
C4—C3—H3	109.2	C14—C15—C16	120.57 (13)
C2—C3—H3	109.2	C14—C15—H15	119.7
C5—C4—C9	116.84 (9)	C16—C15—H15	119.7
C5—C4—C3	121.98 (9)	C15—C16—C11	120.62 (12)
C9—C4—C3	121.13 (9)	C15—C16—H16	119.7
O1—C5—C4	122.22 (9)	C11—C16—H16	119.7
O1—C5—C6	114.71 (9)	O3—C17—H17A	109.5
C4—C5—C6	123.04 (9)	O3—C17—H17B	109.5
C7—C6—C5	117.91 (10)	H17A—C17—H17B	109.5
C7—C6—H6	121.0	O3—C17—H17C	109.5
C5—C6—H6	121.0	H17A—C17—H17C	109.5
O3—C7—C6	123.90 (10)	H17B—C17—H17C	109.5
O3—C7—C8	114.69 (9)	O4—C18—H18A	109.5
C6—C7—C8	121.41 (9)	O4—C18—H18B	109.5
C9—C8—C7	118.96 (9)	H18A—C18—H18B	109.5
C9—C8—H8	120.5	O4—C18—H18C	109.5
C7—C8—H8	120.5	H18A—C18—H18C	109.5
O4—C9—C8	123.60 (9)	H18B—C18—H18C	109.5
			
C5—O1—C1—C2	−44.61 (12)	O3—C7—C8—C9	−178.85 (9)
O1—C1—C2—C3	63.83 (11)	C6—C7—C8—C9	1.05 (15)
O1—C1—C2—C10	−170.72 (8)	C18—O4—C9—C8	−5.74 (14)
C1—C2—C3—O2	73.15 (10)	C18—O4—C9—C4	174.43 (8)
C10—C2—C3—O2	−50.27 (11)	C7—C8—C9—O4	179.55 (9)
C1—C2—C3—C4	−48.90 (11)	C7—C8—C9—C4	−0.63 (15)
C10—C2—C3—C4	−172.32 (8)	C5—C4—C9—O4	179.95 (8)
O2—C3—C4—C5	−100.13 (11)	C3—C4—C9—O4	−2.56 (13)
C2—C3—C4—C5	19.23 (12)	C5—C4—C9—C8	0.11 (14)
O2—C3—C4—C9	82.51 (11)	C3—C4—C9—C8	177.60 (9)
C2—C3—C4—C9	−158.13 (9)	C1—C2—C10—C11	175.01 (9)
C1—O1—C5—C4	12.11 (13)	C3—C2—C10—C11	−62.68 (12)
C1—O1—C5—C6	−169.70 (9)	C2—C10—C11—C12	−88.26 (12)
C9—C4—C5—O1	178.06 (9)	C2—C10—C11—C16	89.45 (12)
C3—C4—C5—O1	0.59 (14)	C16—C11—C12—C13	−0.11 (16)
C9—C4—C5—C6	0.02 (14)	C10—C11—C12—C13	177.66 (10)
C3—C4—C5—C6	−177.45 (9)	C11—C12—C13—C14	−0.46 (17)
O1—C5—C6—C7	−177.79 (9)	C12—C13—C14—C15	0.56 (18)
C4—C5—C6—C7	0.38 (15)	C13—C14—C15—C16	−0.10 (18)
C17—O3—C7—C6	−5.93 (14)	C14—C15—C16—C11	−0.48 (17)
C17—O3—C7—C8	173.97 (9)	C12—C11—C16—C15	0.57 (16)
C5—C6—C7—O3	178.97 (9)	C10—C11—C16—C15	−177.19 (10)
C5—C6—C7—C8	−0.92 (15)		

### Hydrogen-bond geometry (Å, °)

**Table:**

*D*—H···*A*	*D*—H	H···*A*	*D*···*A*	*D*—H···*A*
O2—H2O···O1^i^	0.95 (1)	1.93 (1)	2.8366 (15)	158.(2)

Symmetry codes: (i) −*x*, *y*+1/2, −*z*+1/2.
